# Research progress on the molecular structure, function, and application in tumor therapy of zinc transporter ZIP4

**DOI:** 10.7150/ijbs.102460

**Published:** 2024-11-04

**Authors:** Haijun Guo, Shaohua Wang, Hui Zhang, Jie Li, Chao Wang, Zhikun Liu, Jun Chen, Kai Wang, Xuyong Wei, Qiang Wei, Xiao Xu

**Affiliations:** 1 Department of Hepatobiliary and Pancreatic Surgery, Affiliated Hangzhou First People's Hospital, Zhejiang University School of Medicine, Hangzhou, 310006, China.; 2 Zhejiang University School of Medicine, Hangzhou, 310058, China; 3 Shaoxing city Keqiao District TCM hospital Medical Alliance General Hospital, Shaoxing, 312000, China.; 4 The Fourth School of Clinical Medicine, Zhejiang Chinese Medicine University, Hangzhou, 310053, China.; 5 Department of Hepatobiliary and Pancreatic Surgery, People's Hospital Affiliated to Hangzhou Medical College (Zhejiang Provincial People's Hospital), Hangzhou, 310024, China.; 6 Institute of Translational Medical, Zhejiang University, Hangzhou, 310006, China.

**Keywords:** ZIP family, ZIP4, Molecular structure, Cancer, Molecular Targets.

## Abstract

ZIP4, a pivotal member of the ZIP family, is the causative gene for the hereditary disorder AE (acrodermatitis enteropathica) in humans, and plays an essential role in regulating zinc ion balance within cells. While research on the molecular structure of ZIP4 continues, there remains a lack of full understanding regarding the stereo-structural conformation of ZIP4 molecules. Currently, there are two hypotheses concerning the transport of zinc ions into the cytoplasm by ZIP4, with some contradictions between experimental studies. Recent investigations have revealed that ZIP4 is involved in tumor growth, metastasis, drug tolerance, and various other processes. Most studies suggest that ZIP4 regulates the malignant biological behavior of tumors through zinc ions as a second messenger: however, latest research has identified that ZIP4 itself binds to Ephrin-B1 to regulate tumor metastasis. This review provides a comprehensive summary of the molecular structure of ZIP4 and its mechanism for transporting zinc ions while also exploring mutual regulation between zinc ions and ZIP4. Furthermore, it summarizes recent research progress on the role of ZIP4 in tumors and discusses its potential as a target for anticancer therapy based on an extensive analysis of research findings. These insights can guide future investigations into the role of ZIP4 in tumors.

## Introduction

Zinc ions play a crucial role as micronutrients in facilitating growth, development, immunity, and various physiological functions 1, 2. Their function is indispensable in controlling cell growth, metabolism, and intercellular communication. Zinc transport proteins tightly control the intracellular levels of zinc ions, including free zinc, serving as a second messenger within cells 1, 2. The human body contains approximately 2-3 grams of zinc, with the majority concentrated in skeletal muscles (around 60%), followed by bones (30%), liver, and skin, while the rest 5% is distributed throughout other tissues 3.

Approximately 3000 proteins are known to bind with zinc, constituting around 10% of the genome, and zinc is recognized for its role in regulating gene expression 4-6. The basal levels of zinc exhibit variation across different cell types, and strict maintenance within normal cellular levels is essential to prevent cellular toxicity 3, 7. ZIP4 belongs to the zinc transporter family known as Zrt- and Irt-like proteins (ZIPs) or solute carrier family 39A (SLC39A) 3. The ZIP family primarily facilitates the uptake of extracellular zinc or controls the release of intracellular zinc from organelles within the cell, such as the endoplasmic reticulum (ER), mitochondria, and Golgi apparatus 3. The ZNT (Zinc Transporter) family, also referred to as SLC30A proteins, present in mammalian cells plays a crucial role in either exporting cytoplasmic zinc or importing it into cellular organelles 8.

The families ZIP and ZNT are essential for regulating the levels of zinc ions in the cytoplasm, contributing to a variety of metabolic processes within cells. These processes encompass cell signaling mediated by cytokines and growth factors, as well as the modulation of cell signaling pathways, receptors, and transcription factors 9. Numerous zinc transport proteins have been demonstrated to be indispensable for cellular functions, with disruption in their function due to gene mutations potentially leading to genetic disorders.

In 2002, chromosome 8q24.3 was identified as the pathogenic region of a rare autosomal recessive hereditary disease, acrodermatitis enteropathies (AE) 10. Following the screening of potential target genes, ZIP4 was identified as the pathogenic gene of AE 11. Mutations in ZIP4 result in reduced uptake of Zinc ions, which is responsible for AE. In recent years, research on ZIP4 has advanced significantly, leading to a better understanding of its molecular structure and related functions. As a key member of the ZIP transporter protein family, ZIP4 plays an essential role at various stages of tumor formation, and therefore continuous efforts are being made to improve our understanding of how it regulates cancer cells. Furthermore, there is strong evidence linking ZIP4 to pancreatic and liver cancers, with significant implications for patient outcomes. Given these findings, ZIP4 shows potential as a target for cancer treatment and a potential biomarker. This review aims to provide a recent literature-based comprehensive overview of the molecular structure and functions of ZIP4, analyze its structural and physiological roles, and explore the mechanisms through which it regulates cancer cells to facilitate further advancements in related research areas.

### Understanding ZIP4 from the structure of the ZIP family

Based on sequence similarities, the ZIP family can be categorized into four subfamilies: ZIP I, ZIP II, gufA, and LIV-1 (Liverpool-1) 12-17. ZIP1, ZIP2, and ZIP3 are classified under the ZIP II subfamily, while ZIP9 is categorized under the ZIP I subfamily. ZIP11 is a member of the gufA subfamily, with the other 9 ZIP proteins falling under the LIV-1 subfamily 17. These 9 transport proteins associated with the estrogen-regulated gene LIV-1 have been provisionally identified in mammals 12. The structure of a typical ZIP transporter protein includes 8 transmembrane domains (TMs), with NH2 and COOH ends located extracellularly 18-22 . Each of them possesses a brief COOH end and a spacious cytoplasmic loop positioned between TM3 and TM4 known as intracellular loop 2 (IL2)^23^, ^24^. This cytoplasmic loop contains a histidine-rich motif that exhibits metal-binding properties 12. Furthermore, individuals belonging to the LIV-1 subgroup exhibit a HEXXH pattern in their TMD5, potentially functioning as a metal-binding site. Their arrangement also features an extracellular domain (ECD) at the NH2 end, a characteristic absent in other ZIP subgroups 12, 17. The ECD is characterized by a strongly preserved proline-alanine-leucine (PAL) pattern, and many members of the LIV-1 subfamily have ECDs that are rich in numerous histidine residues 12, 19. ZIP transporter proteins are typically increased on the cell surface in response to zinc deficiency, except for ZIP5, suggesting a strong link between the activity of these transporters and zinc signal transduction 25-27. However, our understanding of zinc signal transduction is currently unclear.

### Binuclear zinc center (BMC)

Out of the 14 members of the mammalian ZIP family, only ZIP13 from the LIV-1 subfamily has been successfully purified from an insect overexpression system, as bottlenecks hinder the overexpression and purification of other members 28. Prokaryotic ZIP has been utilized in mammalian ZIP studies for accurate biochemical structural examination. Determination of the Structure of Overexpressed BZIP (bacterial ZIP) in Bordetella bronchiseptica by X-ray Crystallography 29. The unique arrangement of 3 + 2 + 3-TM is displayed in the crystal structure of ZIPB. TM1 through TM3 can pivot 180 degrees to align with TM6 through TM8, while TM4 and TM5 are symmetrically linked and surrounded by two 3-TM repeat sequences in the center. This distinctive structure sets BZIP apart from all other transporters 29. The arrangement of crystals in BZIP indicates the existence of an inward-facing shape with a dual zinc ion center during transport, hinting at a potential outward-facing conformation 29, 30. TM4 and TM5, in conjunction with M1 and M2, create a binuclear zinc center (BMC) surrounded by conserved polar amino acid residues. A binuclear zinc center (BMC) is formed by TM4 and TM5 along with several conserved polar amino acid residues, including M1 and M2. BMCs are commonly located at the active sites of metal enzymes or in the soluble regions of metal transporters, with limited instances observed amid transport pathways 31, 32. Analysis of over 17,000 ZIPs reveals the existence of BMCs in numerous ZIPs across different species, underscoring their importance in ZIP operation 30. Mutagenesis and transport experiments with HZIP4 (Human ZIP4) have demonstrated the essential role of M1 in zinc transport, while deletion or substitution of M2 with lysine residues only results in a partial reduction of transport activity. Previous studies on HZIP4 have shown that removal M2 or occupying lysine residues of M2 to eliminate M2 function only reduces but does not completely eliminate zinc transport activity, but alterations to M1 result in loss of transport function. It has been observed that the absence of M2 does not impact the substrate selection by HZIP4, but it is crucial for maintaining HZIP4 activity across a wider range of pH conditions 30. Immersion experiments on BZIP crystals revealed that M1 had a higher affinity for metals than M2. Taken together, these findings suggest that M1 serves as the primary transport site, while M2 may potentially modulate transport activity by influencing the function of M1 or the secondary transport site. The crucial role of M1 in transportation has also been observed in other ZIPs 33-37. However, in some ZIPs, the substitution of the metal-chelating residue on M2 with a lysine residue impedes the binding of the second metal ion. In a study on ZIP2, it was found that the substitution of a tyrosine residue in M2 resulted in a significant alteration in the pKa of the adjacent glutamate residue in M1 35. Examining the role of M2 in different ZIPs could offer further understanding of the molecular mechanisms contributing to the varied functions within the ZIP family. The HEXXHE motif was found in TM5, which is a LIV-1-specific motif. According to comparative computational models of BZIP structures, the HEXXHE motif plays a crucial role in forming the binuclear zinc center 12. BZIP contains multiple conserved zinc-binding locations close to the metal outlet, suggesting their potential involvement in facilitating zinc release into the cytoplasm. Hence, the preserved histidine-rich patterns identified in mammalian TMs close to ZIPs could contain numerous zinc-binding locations that effectively move zinc away from the binuclear zinc core.

In the BZIP structure, the metal transport pathway is visible, and it appears that residue S106 on TM2 serves as the gating residue for the entrance pore 29. In many eukaryotic ZIPs, this position is typically occupied by histidine residues, whose importance has been confirmed by mutagenesis studies 29, 36. Metal-binding amino acids located at the channel entrance in Arabidopsis thaliana, specifically those in the extracellular loop between TM2 and TM3, have been demonstrated to influence substrate specificity 34. A sealed space is created by multiple hydrophobic residues between S106 and BMC to shield metal ions from exposure or hydration during their passage 29. A global conformational change (or significant alteration) is necessary for charged metal substrates to travel more than 8 Å in a hydrophobic setting and reach BMC 36. On the other hand, the route for metal discharge from BMC to the opposite side of the membrane is completely unobstructed and water-filled. In the metal ion release pathway, metal ions are sequentially linked to conserved metal-chelating residues. The combination of these residues and BMC creates a metal relay system that is uncommon in other metal carriers. Based on the location of the histidine-rich fragment in the intracellular loop, it may be involved in the release of metal ions, but this part of the structure has not been analyzed so far. The specific role of histidine residues in the intracellular loop and the metal-binding sites along the pathway for metal release is still not fully understood.

### Understanding ZIP4 through the zinc transport theory of the ZIP family

The arrangement of BZIP's crystal structure shows that the 8 transmembrane segments are divided into two groups: one with TM1, 4, 5, and 6, and the other with the remaining 4 segments. As a classical membrane transporter, it undergoes conformational changes during transport 29. The determined crystal structures indicate that BZIP has an inward-facing conformation, with no open entrance to the extracellular space.

The sieve-like histidine 177 (H177) is located within the metal channel beneath the metal binding site M1, oriented towards the cytoplasm. Metal ion release from the binding site M1 occurs via two conformational changes of histidine 177. Below H177 in the direction of the cytoplasm, there is a loop rich in histidine that connects TM3 and TM4 at the exit site, which could aid in the release of zinc ions 29. BZIP is crystallized in a cubic structure of liposomes, where continuous bilayers could be formed in the cubic conformation of liposomes, providing a better simulation of the biological membranes. A cubic structure that can form continuous bilayer liposomes can better simulate the biofilm, and the crystallization of BZIP in this structure allows for better observation of its conformation. Thus, a native or quasi-native state of the cell with a blocked transport pathway on the outside was observed. A pending issue closely linked to the transportation process is how ZIP carriers change their conformation to reveal BMC in the outer space. Previous researchers have proposed two models. In the first model, when the BMC receives metal ions from the extracellular space, interaction of separated residues in the IFC (inward conformation) leads to the closure of the metal release pathway. The model aligns with an alternating access mechanism, revealing the transport site on one side of the membrane at a time without opening it on both sides simultaneously. The second model features an ion channel-like structure, where significant force places BMC in the middle of pore, allowing both ends to open momentarily and simultaneously to both sides 38. Examination of metal transportation in cells utilizing radioactive compounds or fluorescence dyes sensitive to metals in different eukaryotic ZIPs, such as Escherichia coli, has demonstrated that this mechanism adheres to Michaelis-Menten kinetics 29, 30, 33, 37, 39-45. This evidence supports the carrier model 39. However, studies on recombinant BZIP in protein-liposome complexes suggest that transport is unsaturated and support the channel model 38. Research involving protein-liposome complexes indicates that transportation is an electrogenic process that is passive in nature and aligns with the channel model. Regardless of the specific model used it is essential to remove the barrier separating the extracellular entrance from the BMC. This requires the identification of alternative conformational structures that allow for a complete transport cycle. While uncommon, individuals within the transporter family may utilize different transport mechanisms. The chloride channel family consists mostly of chloride channels, with the rest functioning as Cl^-^/H^+^ antiporters that facilitate the movement of Cl^-^ against its concentration gradient 46. The Cystic Fibrosis Transmembrane Conductance Regulator (CFTR) is a unique member of the ABC transporter superfamily, acting as a passive chloride channel rather than an active carrier. This serves as another illustration of its distinct characteristics. When human CFTR is in an outward conformation (OFC) equivalent to the classical ABC transporter, both sides of the CFTR transport channel open simultaneously 47, 48. This contradicts its very similar structure to other ABC family transporters 49. It is uncertain if a comparable scenario exists within the ZIP family, with the majority of family members utilizing a rotating entry method, and others having an exposed metal release route in the OFC. Therefore, studying corresponding structures in specific functional states can be the key evidence to validate the hypothesized model, but these structures are still unknown.

### Transport dynamics of ZIP family proteins

The structure of ZIP family molecules plays an important supporting role in Zn ion transport, but what is the driving force for their transport of metal ions? Various theories have been suggested for the various members of the ZIP group, sparking debate. Studies indicate that ZIP2, ZIP8, and ZIP14 facilitate the movement of zinc ions alongside bicarbonate. Bicarbonate enhances the transportation of metal ions through these ZIPs 25, 50, 51, and this movement is hindered by the anion transport blocker 4,4'-diisothiocyano-2,2'-stilbenedisulfonic acid (DIDS) 50, 51. Since the transport of ZIP8 and ZIP14 does not depend on the electrochemical gradient between the two sides of the plasma membrane, they are thought to transport electrically neutral complexes (M^2+^: HCO_3_^-^=1:2) 52, 53. Recent research on ZIP2 suggests that while ZIP2 does not transport protons alongside zinc ions 35, 37, shows a preference for low pH, which contradicts with bicarbonate co-transport theory. A new research report discovered that pH levels do not affect the transport function of the surrounding area contradicting earlier claims that act as a transporter for Zn^+^ and H^+^ ions 30, 54. A recent investigation suggests that partially hydrated metal ions could be the substances carried by BZIP, with water molecules aiding in the discharge of metal ions from BMC 55. The concept relies primarily on the observation that residues located within the IFC appear to be readily identified by hydroxyl radicals originating from water. Therefore, there should be a sufficiently wide channel filled with water to allow partially hydrated metal ions to pass through 55. Additionally, this result may also support the existence of variable conformations. During the zinc binding process, it was noted that the hydrophilic nature of residues located in the center of the transport route (like M99) remained constant, suggesting that the solvent interaction of these residues is not affected by the transporter's conformational state. Initial examination of cells using isotopes revealed that zinc movement through ZIP2 is influenced by time, temperature, and concentration, and can reach a maximum level, with a Km of 3 μM observed in genetically modified K562 leukemia cells 25. ATP, K^+^, and Na^+^ gradients do not impact ZIP2 activity. However, under HCO_3_^-^ treatment, ZIP2 activity notably rises, indicating a greater attraction to Zn^2+^ and the subsequent cadmium ion. This implies a Zn^2+^, HCO_3_^-^ co-transport process 25. ZIP8 plays a role in testicular damage caused by cadmium and helps explain the co-transport mechanism of Mn^2+^-HCO_3_^-^ in ZIP8-stable transfected mouse embryonic fibroblasts 56. Prior research indicates that ZIP8 exhibits a Km value of 0.62 μM for the adsorption of Cd^2+^ and a reduced attraction to Zn^2+^. However, in a later electrophysiological study in ZIP8-transfected Xenopus oocytes, ZIP8 showed a Zn^2+^-HCO_3_^-^ co-transport mechanism 51, with the inward flow of two HCO_3_^-^ for each transported Cd^2+^ (or Zn^2+^), to maintain electrical neutrality. Absorption of Cd^2+^ and Zn^2+^ had a maximum rate (Vmax) of 1.8±0.08 and 1.0±0.08 pmol/oocyte/h, with respective Km values of 0.48±0.08 and 0.26±0.09 μM 52. Numerous metal carriers located on the exterior of mammalian cells could potentially conceal these impacts. While the quantity of disrupting carriers in Xenopus oocytes may not be significant, ensuring proper protein folding and distribution can occasionally hinder the accurate determination of the transport mechanism. In order to tackle this problem, the clean prokaryotic BZIP has been utilized for analyzing the transport mechanism in protein-liposome complexes dynamic manner. Like zinc-specific channels, BZIP facilitates the transport of unsaturated zinc, resulting in charge generation once the zinc concentration reaches 2 mM. Its transportation process aligns with ZIP2 38. Nevertheless, this contradicts previous findings from cell-based charge analysis, indicating that our comprehension of the zinc transportation process in mammalian ZIPs remains ambiguous.

### The ECD region of ZIP4 and ZIP family proteins

Many members of the ZIP family have only a short, less conserved, and often unstructured extracellular domain (ECD). However, for some ZIP members, particularly those in eukaryotes, a relatively conserved, large, and folded ECD has evolved. An example is ZIP4, a key member of the ZIP family which belongs to the LIV-1 subfamilies and is essential for dietary zinc absorption. Studying ZIP4-ECD is important for three main reasons: first, around half of the mutations that lead to the serious genetic disorder AE are found in this area 57; secondly, when there is a lack of zinc, ZIP4-ECD becomes separated from the complete protein indicating involvement in zinc regulation 58; thirdly, the structure of ZIP4-ECD has only been identified in the ZIP4 from the central brown bat 42. Mutagenesis and transport assays have demonstrated that the deletion of ZIP4-ECD leads to a 75% loss of transport activity underscoring the functional importance of ZIP4-ECD 42.

ZIP4-ECD is stabilized by four conserved disulfide bonds 42. It is composed of two distinct subdomains, with 14 α-helices, a domain rich in helices (HRD), and a PAL motif (PCD) -containing domain, unique to the LIV-1 subfamily. Specifically, ZIP4 and ZIP12 exhibit conservation of HRD within the nine members of the LIV-1 subfamily, unlike the other members which do not possess this region. The HRD of ZIP4 is a protein-folding area made up of the first 156 amino acids and consists of nine α-helices, showing a significant amount of α-helical content (73%) 42. Currently, PCD is only found in ZIP4, 5, 6, 8, 10, and 12, but is absent in ZIP7 and ZIP13. Interestingly, ZIP7 and ZIP13 are located on intracellular organelles such as the endoplasmic reticulum and Golgi apparatus 59, 60, suggesting a link between the presence or absence of HRD and PCD with respect to localization among different ZIP members. PCD consists of 130 amino acids and is located at the C-terminus of ZIP4-ECD comprising five α-helices presenting a helix-turn-helix fold. The PAL motif in PCD's longest α-helix plays a crucial role in both dimerization and stability. Every coil in PCD is involved in forming dimer, with the PAL motif positioned at the heart of the dimerization boundary. Despite having a short ECD with a degenerate PAL motif, ZIP13 is still able to form a dimer, indicating that PCD may not play a significant role in dimer formation for this protein. Additionally, PCD includes a disorganized histidine-rich loop that may be involved in detecting, moving, or storing metals. A recent study showed that the structure of PrPC (prion protein) is similar to the cysteine-rich core (CFC) domain in PCD, so the cellular PrPC may have originated from ZIP about 500 million years ago 61. Furthermore, the PAL motif in PrPc exhibits a similar dimerization structure as ZIP4-ECD 23, 29, 61.

### Regulation of IL2 in the ZIP4 and ZIP family proteins

The IL2 (intracellular loop 2) of ZIP family members is characterized by its long and rich histidine content. Even within the same subfamily or among IL2 sequences from closely related homologs in different species, the conservation is remarkably low. Structural predictions suggest an unstructured nature of IL2 in the BZIP family, which has been confirmed by actual crystallographic studies. Notably, when zinc ions are present at sub-molar concentrations 62, the hydroxyl radical labeling of IL2 significantly decreases, indicating a direct binding of zinc ions to IL2 in BZIP.

Extensive studies have been conducted on the structure and function of IL2 in HZIP4. Nuclear magnetic resonance (NMR) studies have shown that the IL2 peptide from HZIP4 exists in a disordered state 63. Additional research on partially hydrolyzed proteins from purified full-length HZIP4 has supported the highly dynamic characteristics of IL2 64. NMR experiments with zinc titration have shown that histidine amino acids in IL2 have a strong binding affinity to zinc ions at the millimolar level. However, the exact mode of zinc-binding remains elusive, suggesting a dynamic zinc-binding site undergoing rapid equilibria between different conformations 63. These findings suggest an important role for IL2 as a zinc ion sensor in zinc ion-induced ZIP4 endocytosis and degradation. In this mechanism, an increase in cytoplasmic zinc ion concentration saturates the zinc-binding site of IL2, triggering the unique lysine residue ubiquitination in IL2 and subsequent proteasomal degradation.

For Arabidopsis thaliana IRT1 (AtIRT1), a distinct mechanism has been suggested. In the presence of high levels of non-iron transition metal ions, histidine residues grouped in IL2 attach to non-substrate metal ions in plants. Afterward, IL2 is phosphorylated by the CIPK23 enzyme, leading to the polyubiquitination of two lysine sites in IL2 65. Low micromolar concentrations of zinc ions induce the endocytic degradation of HZIP4 in addition to ubiquitination. In this different method of post-translational regulation, the zinc sensor function of the transport site causes a change in the conformation of the BZIP, affecting the conformation of the IL2 “LQL” motif and enabling recognition by the endocytic machinery 64. Therefore, HZIP4, functioning as a receptor, plays a role in both transport and sensing. AtIRT1 65 and ScZrt1/2(Saccharomyces cerevisiae) 24 are also referred to as receptors, suggesting that numerous ZIPs have properties of receptivity, similar to nutrient transporters 24.

The HxH "motif is located in the extracellular loop between α2 and α3 of mouse ZIP4, where it plays a role in regulating zinc ion-mediated ZIP4 endocytosis 66. The "LQL" motif is exclusive to ZIP4 and ZIP12, displaying high conservation among ZIP4 homologs but not among other members of the ZIP family, indicating its specific functionality for these transporters. In contrast, ZIP1 utilizes a standard dileucine pattern in IL2 to facilitate endocytosis 67, while a similar sequence in IL2 of HZIP4 does not serve this function 68. Additionally, IL2 also serves as a regulator of transport activity. Phosphorylation of serine residues (S275 and S276) within the IL2 significantly enhances the transport activity of human ZIP7, resulting in the release of a high quantity of zinc ions from the endoplasmic reticulum 69. Many phosphorylation sites have been discovered in IL2 from different human ZIPs in proteomic studies 70, and although they are abundant, their biological significance still needs further clarification. A recent study found that the peripheral membrane protein Ephrin-B1 inhibits the transport activity of AtIRT1 through interaction with IL2 71.

In conclusion, IL2 demonstrates a wide range of functions and undergoes various post-translational modifications, playing a crucial role in regulating ZIP4 transport activity and endocytic degradation. However, there is currently insufficient structural and biochemical research on the mechanisms described above.

### ZIP4's unique structure among ZIP family members

ZIP4 differs in structure and function from other members of the ZIP family. The ZIP4 protein stands out as the most distinctive member among ZIP transporters, serving as the sole carrier found on the top surface of intestinal epithelial cells, mainly responsible for absorbing zinc from the diet 27, 62. Mutations in the ZIP4 gene that decrease zinc absorption are responsible for causing the uncommon autosomal recessive condition known as Acrodermatitis Enteropathica (AE) 57. Fifteen missense mutations linked to AE have been found in ZIP4, with seven of them situated in the extracellular domain (ECD), highlighting the importance of this specific area 58, 72. Some mutation sites and types are shown in Table 1. The structural characteristics of the ECD in the ZIP family have been primarily elucidated through the crystal structure of mammalian ZIP4-ECD 42. Consequently, ZIP4 exhibits unique structural features in addition to the shared characteristics of the ZIP family. The ECD comprises two distinct subdomains: a helical-rich domain and a domain containing the PAL motif. Four disulfide bonds stabilize the short loop and connect these subdomains. The research explains how the two extracellular domains come together to create a dimer with the PAL motif in the middle. In murine models, specific AE mutations that eliminate the first and fourth disulfide bonds downregulates ZIP4 glycosylation, highlighting the critical importance of these disulfide bonds in ZIP4 folding 42.

Dempski and his team studied the functions of the cytoplasmic loop (IL2) located between transmembrane domains 3 and 4 (TM3 and TM4) of HZIP4 in humans 73 through site-specific mutagenesis, examination of metal-binding affinity, and analysis using X-ray absorption spectroscopy. The researchers revealed the sequential binding of two Zn^2+^ ions to separate sites in the cytoplasmic loop, attributed to variations in zinc affinity at these locations. Initially, one Zn^2+^ ion binds to the cysis3 site with micromolar affinity, followed by a second Zn^2+^ ion that binds to the His4 site with weaker affinity 73. This mechanism suggests that the M3 M4 domain has a zinc-sensing role and may participate in regulating the expression levels of ZIP4 on the cell membrane based on zinc concentrations in the cytoplasm 73.

**Figure 1:**
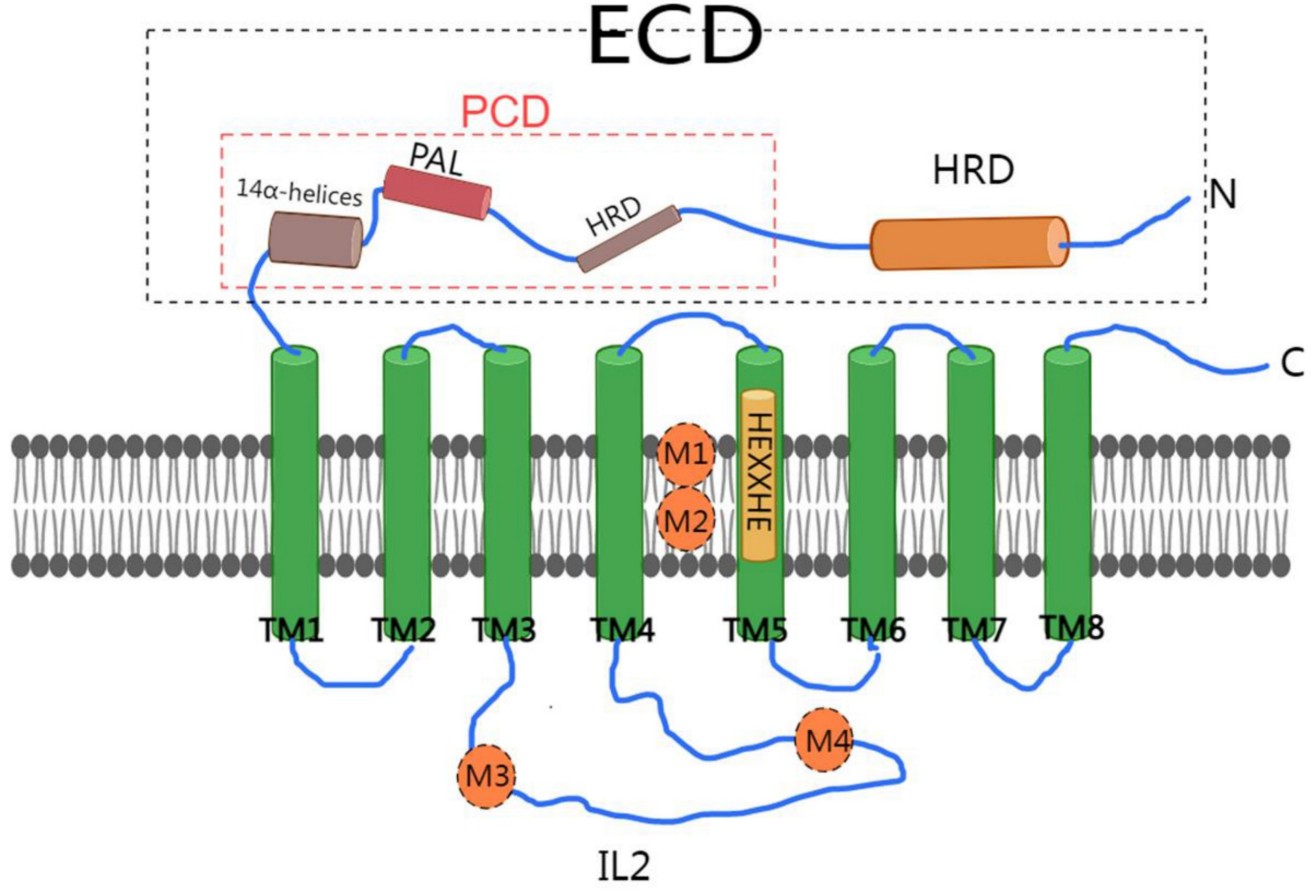
Schematic diagram of ZIP4 structure. ZIP4 consists of 8 transmembrane domains (TM1-TM8). The HEXXHE motif is found in TM5. The extracellular domain (ECD) of ZIP4 is composed of two distinct subdomains, with 14 α-helices, a domain rich in helices (HRD), and a PAL motif (PCD). The cytoplasmic loop (IL2) is located between transmembrane domains 3 and 4 (TM3 and TM4) of HZIP4. M1 and M2, create a binuclear zinc center (BMC). M3 and M4 were in IL2.

In conclusion, we have depicted a schematic diagram illustrating the structure of ZIP4 (Figure 1).

### ZIP4 regulates zinc ions through negative feedback

**Figure 2:**
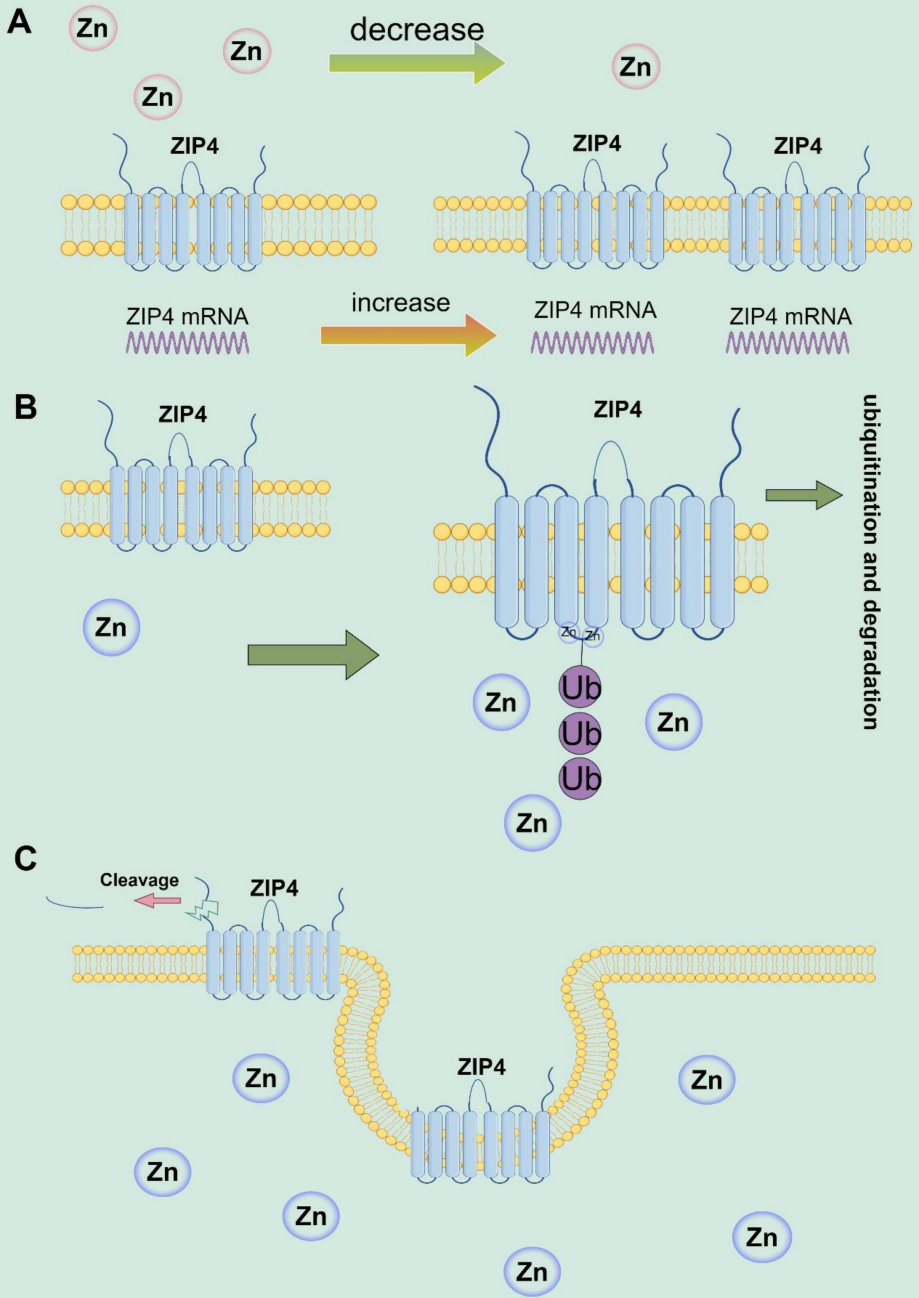
ZIP4 regulates zinc ions through negative feedback. **(A)** In conditions of zinc deficiency, the expression of ZIP4 mRNA and proteins increases in cell membranes. **(B)** When intracellular zinc ion concentration increases, ZIP4 expression decreases through ubiquitination of lysine residues characteristic of IL2 and proteasomal disassembly. **(C)** When intracellular zinc ion concentration increases, ZIP4 reduces its expression at the cell membrane through membrane endocytosis and ECD cleavage.

Studies have revealed the vital function of ZIP4 in zinc transportation, transporters processing, and monitoring 42, 62, 74. Research on HZIP4 and mouse (mZIP4) have elucidated how the cytoplasmic zinc ion concentration regulates ZIP4 surface expression 24. In conditions of zinc deficiency, mZIP4 mRNA expression increases in intestinal epithelial cells and yolk sac cell membranes of mice; conversely, when zinc ions are abundant, mZIP4 expression decreases 27, 75. Additionally, feeding zinc-rich food to zinc-deficient mice results in reduced expression of mZIP4 protein on the cell membrane 27. The high cytoplasmic concentration of Zn^2+^ reduces the expression of mZIP4 on the cell membrane through Zn^2+^-dependent endocytosis 63. According to the study by Kim et al., mZIP4 and HZIP4 proteins accumulate on the cell membrane when zinc is deficient, and ZIP4 endocytosis occurs when the intracellular zinc ion concentration increases to about 1 μmol/L, resulting in reduced zinc ion absorption 63. Additionally, according to Mao et al., the histidine residue-rich bloc in the intracellular domain of ZIP4 promotes ubiquitination and proteasomal disassembly of HZIP4 when cytoplasmic zinc levels are elevated. This mechanism helps to protect against the harmful effects of zinc ions 76. Studies have also discovered that ZIP4-ECD cleavage in intracellular zinc sufficient forms a ZIP4 peptide of approximately 35 kDa. These peptides aggregate as peripheral minor membrane proteins when the remaining ~ 37-kDa ZIP4 COOH terminus is processed and aggregated as essential membrane proteins 77. Two mutations in AE were found to block ECD cleavage, while others reduced it, suggesting that the proteolytic cleavage of ECD plays a crucial role in regulating the zinc homeostasis of ZIP4 62. ECD cleavage was observed in ZIP4, ZIP6, and ZIP10 78. The studies demonstrate that ZIP4 regulates the concentration of intracellular zinc ions through a feedback mechanism. When extracellular zinc ion concentration is too low, ZIP4 aggregates on the membrane surface. Conversely, when intracellular zinc ion concentration increases, ZIP4 decreases expression through membrane endocytosis and ECD cleavage, promoting ZIP4 ubiquitination and degradation, thereby reducing zinc ion transport (Figure 2).

### ZIP4 and Growth Development

ZIP4 is essential for the growth and development of embryos. Defects in ZIP4 can result in an uncommon autosomal recessive metabolic condition called acrodermatitis enteropathica (AE), which is characterized by a lack of zinc, especially in babies 11, 57. Moreover, ZIP4 is crucial for zinc absorption in the gut and is vital for the development and maintenance of Paneth cells within the intestinal epithelium 79. Additionally, ZIP4 enhances the growth of intestinal epithelial cells 79. Mice without ZIP4 show damage to the villus structure, highlighting the importance of ZIP4 in maintaining the structural integrity of the intestinal epithelium. Homozygous mice with a ZIP4 mutation have a high mortality rate during embryonic stages, while mice with a heterozygous ZIP mutation are prone to zinc deficiency, leading to issues such as brain and eye abnormalities and slowed growth 64. Zinc ions are actively transported and primarily absorbed in the duodenum, jejunum, and ileum through ZIP4 65. In the skin, ZIP4 is crucial for maintaining homeostasis in the human epidermis by regulating zinc-dependent ΔNp63 activity 80. In summary, ZIP4 plays a significant role in proliferating small intestinal epithelial cells, maintaining intestinal integrity, and regulation zinc absorption in the intestine. The lack of ZIP4 during embryonic development results in developmental abnormalities and hereditary diseases.

### The Role of ZIP4 and Zinc Ions in Tumor Growth

Previous studies have indicated an increased expression of ZIP4 in human pancreatic cancer tissues and cell lines, which promotes tumor growth. Reduced levels of ZIP4 inhibit the growth of pancreatic cancer cells by decreasing cyclin D1, a target of the cAMP-response element binding protein (CREB)/miR-373/PHLPP2 and CREB/IL-6/STAT3 pathways 66, 81. Overexpression of ZIP4 activates both pathways, leading to a significant increase in pancreatic cancer cell proliferation. Similarly, elevated expression of ZIP4 is observed in liver cancer, where it facilitates mitosis 82. Knocking out ZIP4 in ovarian cancer significantly reduces the activity of ovarian cancer stem cells, including proliferation, anti-apoptosis, colony and spheroid formation, and drug resistance 83, 84. An additional research study indicates that ZIP4 stimulates the development of oral squamous cell carcinoma when combined with zinc within cells 85. In oral squamous cell carcinoma, ZIP4 is upregulated and its absence leads to reduced cell proliferation 85.

The high expression of ZIP4 indicates elevated zinc levels in pancreatic cancer 86. In a subcutaneous xenograft model in nude mice, it was found that zinc content increased by 80% in tumors transplanted with stable MIA-ZIP4 cells compared to the normal group 87. Additionally, upregulation of ZIP4 expression enhances zinc influx, promoting the tumorigenicity of triple-negative breast cancer 88. However, experiments have shown that zinc levels are significantly reduced in early pancreatic cancer compared to normal or benign pancreatic tissue 16. In pancreatic intraepithelial neoplasia (PanIN) and malignant tumors, the decrease in zinc levels is attributed to the downregulation of Ras-responsive element-binding protein 1 (RREB-1) and the silencing of ZIP3 16, 89, 90. Meanwhile, studies have found that pancreatic cancer cells are sensitive to high concentrations of zinc. Exposure to normal levels of zinc (0.01-0.5 mM) results in cell death for pancreatic cancer (PC) cells 91. Zinc is essential for cell function, growth, proliferation, and metabolism. Healthy cells have developed mechanisms to regulate zinc levels and avoid negative effects from excessive amounts of zinc. However, malignant cells lose these normal protective conditions. Therefore, as normal cells transform into cancer cells, their requirement for zinc ions and tolerance to zinc toxicity continuously change. Nevertheless, it is believed that ZIP4 and zinc ions collectively promote tumor cell proliferation.

### ZIP4 promotes tumor invasion and metastasis

Numerous studies have indicated that ZIP4 can promote the invasion and metastasis of pancreatic cancer cells 87. ZIP4 induces the expression of YAP1 in pancreatic cancer by activating the miR-373-LATS2 pathway, thereby increasing the expression of ITGA3 and promoting cell adhesion 92. ZIP4 induces the expression of ZEB1, a key transcription factor of EMT (Epithelial-Mesenchymal Transition) in pancreatic cancer, by phosphorylating STAT3, thereby promoting the stemness, invasion and metastasis of pancreatic cancer 93, 94. ZIP4 also induces the expression of ZEB1, suppressing the expression of ZO-1 and Claudin-1 to induce migration and invasion in pancreatic cancer 95. In liver cancer, our research suggests that ZIP4 interacts with Ephrin-B1 and regulates the ubiquitination of Ephrin-B1 to affect the downstream Wnt5A/JNK/ZEB1 signaling pathway, thereby promoting EMT transformation and invasion and metastasis of HCC 96. Additionally, other studies have found that ZIP4 promotes EMT in NSCLC (Non-small cell lung cancer) by activating the Snail-calcium pathway 97, 98. Furthermore, increased expression of ZIP4 in the C666-1.77 cell line of nasopharyngeal carcinoma triggers the PI3K/AKT signaling pathway, leading to EMT 99. In conclusion, ZIP4 can promote EMT and induce tumor invasion and metastasis through ZEB1 and ITGA3.

### ZIP4 hinders tumor therapy

**Figure 3:**
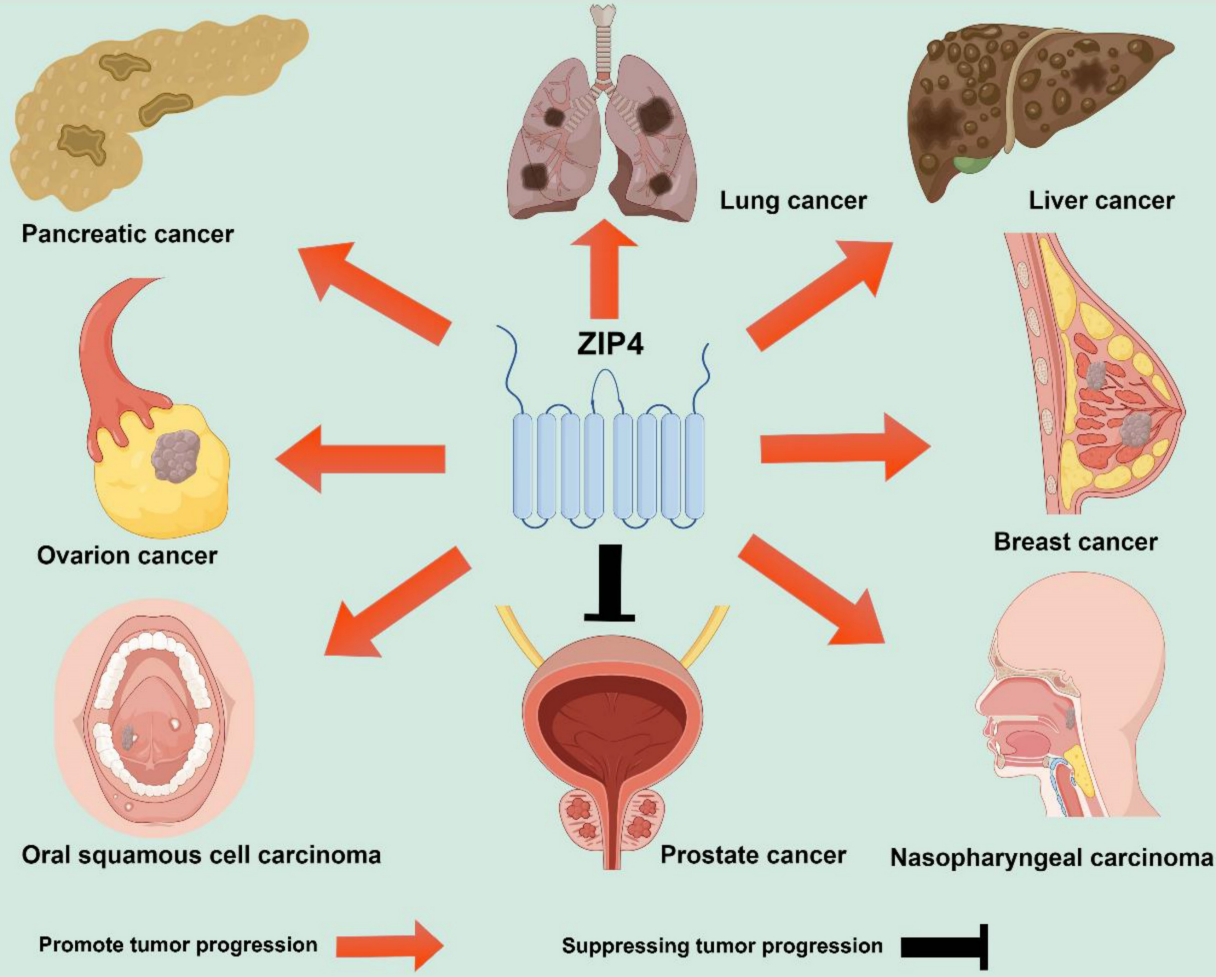
ZIP4 regulates pancreatic carcinoma, lung carcinoma, liver carcinoma, ovarian carcinoma, breast carcinoma, oral squamous cell carcinoma, prostate carcinoma and nasopharyngeal carcinoma. ZIP4 promotes pancreatic cancer, lung carcinoma, liver carcinoma, ovarian carcinoma, breast carcinoma, oral squamous cell carcinoma and nasopharyngeal carcinoma but inhibits prostate cancer. The orange arrow indicates the promotion of tumor progression while black indicates inhibition of tumor progression.

The overexpression of ZEB1 was found to activate the ITGA3/ITGB1/α3β1 signaling pathway and the c-JNK pathway, leading to a reduction in the expression of gemcitabine transporter 94. This mechanism explains how ZIP4 promotes resistance to gemcitabine in pancreatic cancer 94. ZIP4 is also involved in the development of cachexia associated with pancreatic cancer by facilitating the secretion of HSP70 and HSP90 via extracellular vesicles, ultimately promoting muscle wasting 100. In pancreatic cancer, CircANAPC7 plays a role in reducing muscle wasting by interfering with the ZIP4/miR-373 pathway through the STAT5/TGFβ signaling pathway 101. Furthermore, ZIP4 not only promotes the progression of pancreatic cancer through the CREB-activated syndecan 1 (SDC1) and dynamin 2 (DNM2) pathway but also promotes the glycogen synthase kinase 3β(GSK3β)/necrosis factor superfamily member 10 (TRAIL) pathway to drive muscle consumption, thereby aggravating cachexia 102. Similarly, interference with ZIP4 expression leads to a shift towards an epithelial phenotype in non-small cell lung cancer, resulting in decreased levels of cancer stem cell (CSC) markers and increased responsiveness to cisplatin 98. High levels of ZIP4 expression in high-grade serous ovarian cancer (HGSOC) have been shown to enhance resistance to cisplatin and paclitaxel chemotherapy 83. Additionally, another study indicated that inhibiting ZIP4 enhances radiation-induced apoptosis in nasopharyngeal carcinoma C666-1 cells both *in vitro* and *in vivo* 99. Therefore, targeting ZIP4 combined with radiation therapy may represent an effective new approach for treating nasopharyngeal carcinoma 99. These findings underscore the crucial role of ZIP4 in the chemotherapy and radiotherapy sensitivity of malignant tumors, suggesting that inhibitors targeting ZIP4 could significantly improve the therapeutic outcomes of tumors. The involvement of ZIP4 in the regulation of a variety of tumors is shown in Figure 3.

## Discussion

The zinc ions and the zinc transporter family have crucial importance in various biological functions, encompassing cell metabolism and zinc signal transduction. This regulatory role extends across fundamental cellular activities and various processes in the development of diseases. Currently, the structure characteristics of ZIP4 and its family remain ambiguous. Upon amalgamating multiple studies, we can delineate the primary structural domains and crucial motifs of ZIP4. However, extensive research is still required to confirm and ascertain the specific functions of these elements. Among the ZIP family members, ZIP4 is one of the most extensively studied in terms of structure and function in mammals. The crystal structure of ZIP4's extracellular domain (ZIP4-ECD) has been revealed, providing insight into this aspect of the ZIP family's structure. From studies on ZIP4, it is established that there are two zinc ion binding sites in IL2, serving as zinc ion sensors. IL2 plays a crucial bridging role in the negative feedback regulation between ZIP4 and zinc ions.

The mechanism of zinc ion transport by ZIP4 is still controversial, with ongoing debates on whether the ion channel mode or carrier-mediated counter-gradient transport predominates. If it operates in a mode, what are the structural and conformational changes? Is there an associated energy consumption? Alternatively, if it functions as an ion channel mode, how does the ion channel open? Is zinc ion transport facilitated by electrically neutral Zn^2+^/2HCO_3_^-^ ions, or is it through Zn^2+^/H^+^ cotransport? All these questions and inquiries necessitate further investigation. The possibility of a mixed mode among ZIP family members adds complexity and contributes to conflicting results that cannot be reconciled. To elucidate the specific transport mechanism of ZIP4, individual studies on the ZIP4 protein are imperative, results from other members should not serve as substitutes.

**Figure 4:**
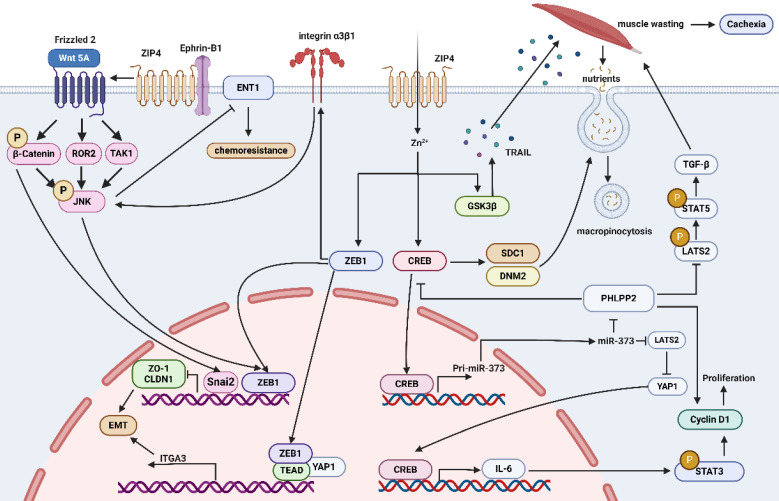
Mechanisms by which ZIP4 regulates tumors. (1) The pathways of CREB/miR-373/PHLPP2/cyclin D1 and CREB/IL-6/STAT3/cyclin D1 promote cell proliferation and tumor progression. (2) STAT3/ZEB1/ZO-1/Claudin-1, miR-373-LATS2-YAP1/ITGA3 promote EMT. (3) ZEB1/ITGA3/ITGB1/α3β1/c-JNK, CREB-miR-373-PHLPP2 promote tumor drug resistance. (4) Ephrin-B1/Wnt5A/JNK/ZEB1 promotes EMT. (5) GSK3β/TRAIL and CREB/SDC1/DNM2 promote macropinocytosis and muscle wasting.

ZIP4 plays a crucial role in promoting tumor growth. Overexpression of ZIP4 is observed in many tumors, possibly due to increased demand for zinc ions by tumor cells and its involvement in the regulatory mechanisms of tumor cells. Current research indicates that the regulatory mechanisms of ZIP4 in tumors primarily involve pathways: 1) CREB/miR-373/PHLPP2/cyclin D1 and CREB/IL-6/STAT3/cyclin D1 promoting cell proliferation and tumor progression; 2) STAT3/ZEB1/ZO-1/Claudin-1, miR-373-LATS2-YAP1/ITGA3 promoting EMT; 3) ZEB1/ITGA3/ITGB1/α3β1/c-JNK, CREB-miR-373-PHLPP2 promoting tumor drug resistance; 4) Ephrin-B1/Wnt5A/JNK/ZEB1 promoting EMT; 5) GSK3β/TRAIL and CREB/SDC1/DNM2 promoting macropinocytosis and muscle wasting (Figure 4). From the above mechanisms, the transcription factor CREB appears to be the upstream protein for all mechanisms. Therefore, how does ZIP4, as a membrane protein, regulate transcription factors? Previous studies believed that ZIP4 conveys regulatory information through zinc ions as messengers. However, it has been discovered that Ephrin-B1 interacts with IL2 to inhibit AtIRT1 transport activity, and the mechanism is very similar to that we found that ZIP4 binds Ephrin-B1 to inhibit its ubiquitination and thereby promote EMT in HCC. We also found that ZIP4 interacts with the extracellular domains of Ephrin-B1. Based on these findings, it can be speculated that IL2 or ECD of ZIP4 may bind to the extracellular domains of other membrane proteins, exerting regulatory effects. Therefore, it is likely that the IL2 or ECD region of ZIP4 contains structures that can bind to other membrane proteins, thus exerting regulatory effects through them and influencing the tumor microenvironment, promoting tumor growth and metastasis. We believe that ZIP4's regulatory role in zinc ions may not be the sole method of regulating cell activities. ZIP4 and its family members are likely to be oncogenes themselves, capable of independently promoting the occurrence and development of cancer cells regardless of zinc ion effects. Therefore, targeted drugs against ZIP4 and its family may not only inhibit zinc ion absorption but also kill cancer cells, making them a promising therapeutic target. Of course, our speculations need further validation through extensive research. Still, we hope this review provides fellow researchers studying ZIP family proteins with a usable perspective for translating ZIP4 and ZIP family research into clinical drug studies for treating various diseases.

## Conclusions

Current studies have only revealed a part of the molecular structure and some specific functions of each part of the molecular structure of ZIP4. Further improvement in the purification method for molecular crystallization, complete purification, and crystallization of mammalian and human ZIP4 is essential for research on its molecular structure. Based on this, numerous experiments are needed to investigate the specific functions of various parts of the ZIP4 molecular structure to truly understand its mechanism of action of ZIP4. Previous studies on zinc ion transport by ZIP4 and the negative feedback regulation mechanism still require further evidence to address the contradictory findings. In tumors, ZIP4 can play a regulatory role through various signaling pathways. Zinc ions may not be the sole means of signal transduction, subsequent studies are needed to verify whether ZIP4 binds other molecules to directly deliver regulatory signals. To summarize the current research results of ZIP4 in tumors, it could promote growth and development in a wide variety of tumors while also inhibiting tumor invasion and metastasis, as well as improving tumor treatment outcomes. Therefore, there is an expectation that drug development targeting ZIP4 will become a new approach for targeted therapy against tumors.

## Figures and Tables

**Table 1:** ZIP4 mutation sites associated with Acrodermatitis Enteropathica pathogenesis 57.

No.	Type of mutation	Location of mutation	Acrodermatitis enteropathica
1	Deletion	Exon 7 (1223-1227 del CCGGG)	Yes
2	Deletion	Exon 5 (968-971 del AGCT)	Yes
3	Mutations	IVS1-19 Gly→Ala	probably
4	Mutation	Exon 3 (599 C→T)	Yes
5	Mutation	Exon 10 (1576 G→A)	Yes
6	Mutation	Exon 6 (1120 G→A)	Yes
7	Mutations	526Gly→Arg	probably
8	Mutations	374Gly→Arg	probably

Abbreviations: Del, Deletion; Gly, glycine; Ala, alanine; Arg, arginase. G, T, C and A are base pairs of DNA.

## Abbreviations

AE: acrodermatitis enteropathica
ER: endoplasmic reticulum
ZNT: Zinc Transporter
TMDs: transmembrane domains
IL2: intracellular loop 2
ECD: extracellular domain
PAL: proline-alanine-leucine
BMC: Binuclear zinc center
BZIP: bacterial ZIP
HZIP4: Human ZIP4
NMR: Nuclear magnetic resonance
mZIP4: Mouse ZIP4
IFC: inward conformation
OFC: outward conformation
HRD: a domain rich in helices
PCD: a PAL motif
PrPC: prion protein
CFC: the cysteine-rich core
NMR: Nuclear magnetic resonance
AtIRT1: Arabidopsis thaliana IRT1
ScZrt1/2: Saccharomyces cerevisiae
NSCLC: Non-small cell lung cancer
EMT: Epithelial-Mesenchymal Transition
CSC: cancer stem cell
HGSOC: high-grade serous ovarian cancer
